# Synthesis of truncated analogues of the *i*NKT cell agonist, α-galactosyl ceramide (KRN7000), and their biological evaluation

**DOI:** 10.1016/j.bmc.2010.11.032

**Published:** 2011-01-01

**Authors:** Natacha Veerapen, Faye Reddington, Mariolina Salio, Vincenzo Cerundolo, Gurdyal S. Besra

**Affiliations:** a School of Biosciences, University of Birmingham, Edgbaston, Birmingham B15 2TT, UK; b The Weatherall Institute of Molecular Medicine, University of Oxford, John Radcliffe Hospital, Oxford OX3 9DS, UK

**Keywords:** α-Galactosyl ceramide, OCH, Th1, Th2, Sphingosine

## Abstract

Stimulation of *i*NKT cells by α-galactosyl ceramide (α-GalCer), also known as KRN7000, and its truncated analogue OCH induces both Th1- and Th2-cytokines, with OCH inducing a Th2-cytokine bias. Skewing of the *i*NKT cells’ response towards either a Th1- or Th2-cytokine profile offers potential therapeutic benefits. The length of both the acyl and the sphingosine chains in α-galactosyl ceramides is known to influence the cytokine release profile. We have synthesized analogues of α-GalCer with truncated sphingosine chains for biological evaluation, with particular emphasis on the Th1/Th2 distribution. Starting from a common precursor, d-lyxose, the sphingosine derivatives were synthesised via a straightforward Wittig condensation.

## Graphical abstract

**Figure:**
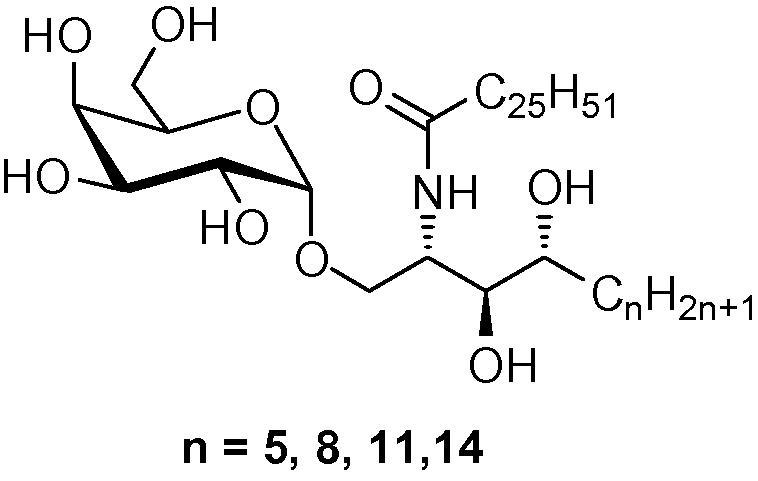

## 1 Introduction

**Figure 1:**
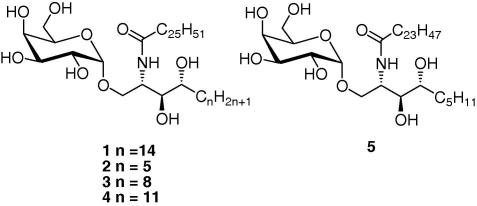
CD1d agonist KRN7000, OCH, and analogues.

The distinctive class of T cells, known as invariant Natural Killer T (*i*NKT) cells, display characteristics of both T cells and NK cells and play a crucial role in diverse immune responses and other pathologic conditions.^1^ The synthetic glycolipid α-galactosyl ceramide (α-GalCer),^2^ known as KRN7000 (**1**) (Fig. 1), is a powerful agonist, which when presented by CD1d, activates *i*NKT cells to release diverse cytokines, including both Th1- and Th2-cytokines.^3^ Once stimulated, *i*NKT cells also activate other cells such as dendritic cells, T cells and B cells.^4^ It is believed that the release of proinflammatory Th1 cytokines such as interferon-γ (INF-γ) may contribute to antitumour and antimicrobial functions while that of immunomodulatory Th2-cytokines such as interleukin 4 (IL-4) may help alleviate autoimmune diseases^5^ such as multiple sclerosis^6^ (MS) and arthritis.^7^ Maintaining the right balance between Th1- and Th2-cytokines is of utmost importance as over activation of Th1 cells or suppression of Th2 ones can lead to autoimmune diseases.^8^

Moreover, skewing of the cytokine release profile towards a Th2 one can help in the treatment of autoimmune and inflammatory conditions.^9^, ^10^ For example, compound **5**, commonly referred to as OCH (Fig. 1), has been shown to protect mice against experimental encephalomyelitis (an animal model for MS) by favouring the release of the Th2-cytokine IL-4 and suppressing the myelin antigen-specific Th1 responses.^6^, ^11^

Crystal structures of both mouse^12^ and human CD1d^13^ have identified the antigen-binding site as consisting of two channels or pockets; A′ and F′, lined with hydrophobic residues. While the A′ channel can accommodate an alkyl chain consisting of up to 26 carbons (the acyl chain in α-GalCer), the F′ channel can accommodate an alkyl chain of 18 carbons (the sphingosine chain in α-GalCer).^13^, ^14^ The stability of the bound glycolipid/CD1d complex and in turn the binding affinity of the latter to T cell receptors (TCR) are believed to largely influence the immunological response.^6^, ^14^ The apparent skewing towards a Th2 response in the case of OCH (**5**) is allegedly due to a less stable bound CD1d complex resulting in a less prolonged TCR stimulation.^6^, ^14^, ^15^ α-Galactosyl ceramides with variations in the acyl chain have been extensively studied, and have also exhibited similar effects on the cytokine release profile.^9^ However, the properties of analogues of α-GalCer with variations in the sphingosine chain, such as OCH (**5**) have yet to be fully explored.

α-GalCer and its counterparts have proved to be and remain invaluable tools in understanding the functioning of CD1d and NKT cells in a wide range of immune responses. Specifically, compounds such as OCH (**5**), that are able to alter the polarisation of Th1 or Th2, have potential therapeutic values for certain diseases. As such, we have synthesised compounds **2**, **3** and **4** (Fig. 1), with varying phytosphingosine chain lengths consisting of 9–15 carbons, to investigate their effect on the Th1/Th2 balance as well as to study their overall biological properties.

## 2 Results and discussion

Various methods, including dihydroxylation reactions^16^ and Sharpless asymmetric epoxidation^17^ have been described in the literature for the synthesis of sphingolipids.^18^ Yet, the preparation of such compounds remains nontrivial. In contrast to many reported syntheses, the strategy employed by Lin et al.^19^ is quite concise and affords a relatively high yield of the final phytosphingosine from the readily available d-lyxose. Their method is particularly attractive as d-lyxose already possesses the required stereogenic centres and an S_N_2 displacement of a suitable leaving group at C-4 allows the introduction of the amine functionality in the molecule. It is noteworthy that Plettenburg et al.^20^ also used a similar strategy, involving conversion of their substrate to phytosphingosine via a Wittig condensation, in their reported synthesis.

**Scheme 1:**
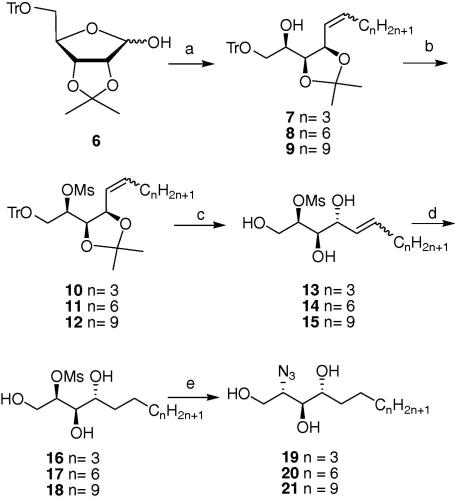
Reagents: (a) *n*-BuLi, THF, phosphonium salts (C_4_H_9_PPh_3_^+^I^−^, C_7_H_15_PPh_3_^+^Br^−^, C_10_H_21_PPh_3_^+^Br^−^; (b) MsCl, pyridine, CH_2_Cl_2_, quant; (c) HCl, MeOH/CH_2_Cl_2_; (d) H_2_, Pd–BaSO_4_, THF; (e) NaN_3_, DMF.

We have therefore adapted both these methods to synthesise our analogous sphingosines. We first embarked on the synthesis of the appropriate phosphonium salts required for the chain extending Wittig olefination reaction. This was easily achieved by refluxing triphenylphoshine with the corresponding alkyl halides in toluene overnight. For the C-9, C-12, and C-15 phytosphingosine chain lengths, 1-iodobutane, 1-bromooctane and 1-bromodecane were used, respectively. Once isolated, they were resuspended in THF and treated with *n*-BuLi at −78 °C to generate the Wittig ylids, as described by Plettenburg et al.^20^ Subsequently, the protected lyxose derivative **6**, which was synthesised as previously described,^19^ was condensed with the ylids to afford the desired olefins **7**, **8** and **9** as a mixture of *cis* and *trans* isomers (Scheme 1). In their preparation of the C-18 phytosphingosine, Lin et al.^19^ proceeded to the reduction of the double bond after the Wittig condensation via catalytic hydrogenation using palladium hydroxide (Pd(OH)_2_). However, in our hands, after several attempts the hydrogenation failed to go to completion after prolonged reaction periods. In an attempt to drive the reaction to completion, the catalyst was filtered off and replaced with fresh one. Although the reaction was eventually completed after 48 h, the trityl protecting group was also cleaved, thereby leaving the primary alcohol unprotected. An alternative strategy where the protecting groups would be removed prior to hydrogenation of the double bond was therefore envisaged. Hence, the remaining free secondary hydroxyl group was mesylated by reaction with methanesulfonyl chloride in dichloromethane. The mesyl group acts both as a temporary protecting group and a good leaving group for the following inversion of stereochemistry at this position. Removal of the trityl and acetonide protecting groups by acid treatment then yielded compounds **13**, **14** and **15** in quantitative yields. The reduction of the double bond by hydrogenation, catalysed by 5% palladium on barium sulphate proceeded smoothly to give compounds **16**, **17** and **18**. We substituted the Pd(OH)_2_ with palladium on barium sulphate because the latter is less active and is more suitable for use in the presence of the mesylate group. Finally, the amine functional group was introduced into the molecule by an S_N_2 displacement of the mesylate with sodium azide in DMF. Sphingosine analogues **19**, **20** and **21** were thus obtained in reasonable yields for further use as glycosyl acceptors.

**Scheme 2:**
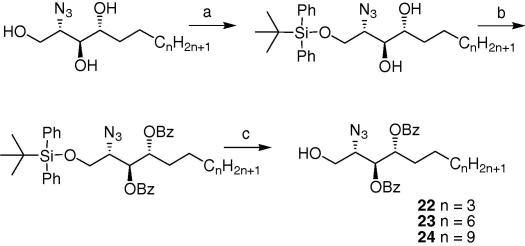
Reagents: (a) TBDPSCl, pyridine, quant; (b) BzCl, Pyr, 86%; (c) TBAF, THF, 80%.

Hence, following the standard conditions described previously^21^ and summarised in Scheme 2, the sphingosine acceptors **22**–**24** were synthesized. Consistent with our previous work, benzoate esters, rather than benzyl ethers, were adopted as protecting groups to circumvent the hydrogenolysis reaction.

**Scheme 3:**
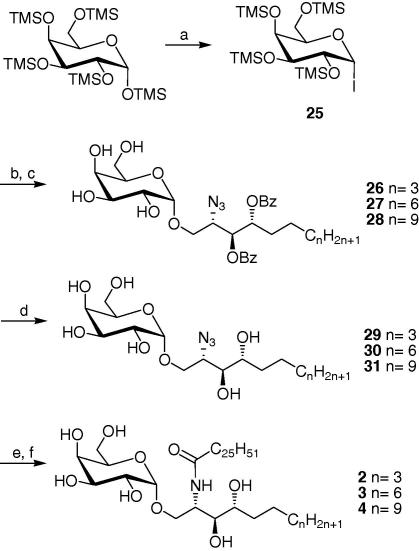
Reagents and conditions: (a)TMSI, CH_2_Cl_2_, 0 °C, quant; (b) TBAI, DIPEA, **22**, **23** and **24**, benzene, rt; (c) Dowex 50WX8-200, MeOH, rt, 62% over two-steps; (d) NaOMe/MeOH, quantitative; (d) H_2_, Pd, MeOH, 80%; (e) C_25_H_51_COCl, THF/NaOAc (1:1), 71%.

With respect to the glycosyl donor, we have previously successfully employed the bulky 4, 6-*O*-di-*tert*-butylsilylene (DTBS) group as α-directing in galactosylation donors.^22^ DTBS ensures the exclusive formation of an α-glycosidic linkage, irrespective of the nature of the acceptor, and remains the protecting group of choice in challenging glycosylation reactions. However with our glycosyl acceptors, we were inclined to adopt Gervay-Hague’s rather simplified glycosylation strategy employing glycosyl iodides and promoted by tetrabutyl ammonium iodide (TBAI).^23^ Indeed, the latter’s group recently reported both excellent stereoselectivity and yields for the synthesis of α-GalCer and other similar compounds.^24^, ^25^ It is hypothesised that the α-selectivity is due to the TBAI-catalysed isomerization of the α-glycosyliodide to the β-anomer.^26^ This strategy represents a straightforward approach to the synthesis of our target compounds given that the donor can be easily obtained in large quantities in contrast to the multi-step preparation of other galactosyl donors. The per-*O*-tetramethylsilyl-α-d-galactopyranosyl iodide **25** was therefore generated by the reaction of the per-*O*-pentamethylsilyl-α-d-galactose with 1 equiv of iodotrimethylsilane^26^ and then added to the respective phytosphingosine acceptors **22**–**24** which were premixed with diisopropylethylamine (DIPEA) and TBAI (Scheme 3). After two days at room temperature, the solvent was evaporated and the TMS protecting groups were removed by treatment with an acidic resin in MeOH. The glycosylated compounds **26**–**28** were obtained as the α-anomer exclusively in good overall yields of over 60%. The formation of the desired α-linkage in compounds **26**–**28** was confirmed by the H-1 and C-1 signals in ^1^H and ^13^C NMR, respectively. Finally, Zemplen’s deprotection of the benzoate protecting groups, followed by hydrogenation of the azido group in methanol provided the amines which were acylated with the fully saturated fatty acid, hexacosanoic acid. This was accomplished by reaction of the corresponding acid chloride with the free amine in a 1:1 mixture of THF and saturated sodium acetate solution. Glycosphingolipds (GSL) **2** (OCH9), **3** (OCH12) and **4** (OCH15) were obtained as white solids after concentration of the organic phase and purification of the residue by flash chromatography.

**Figure 2:**
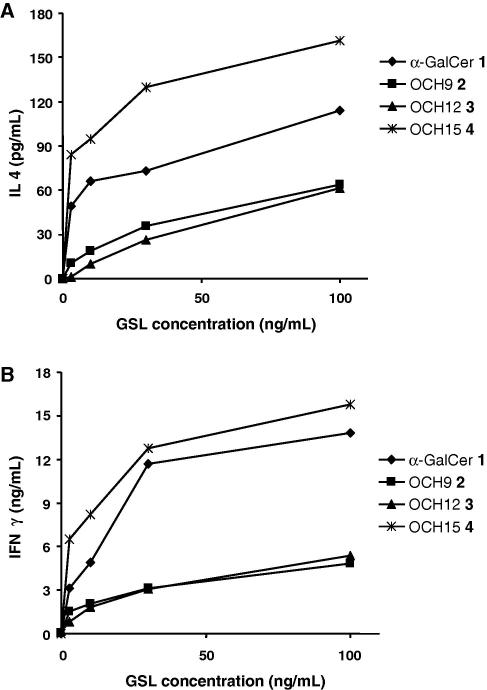
CIR-hCD1d cells were pulsed with GSL and used to stimulate *i*NKT cells. Supernatant was assayed for IL-4 (**A**) and IFN-γ (**B**) by ELISA.

**Figure 3:**
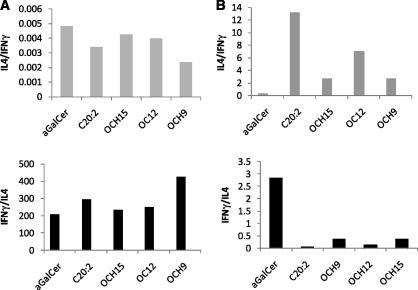
A (upper and lower pannel) IL4/IFNγ and IFNγ/IL4 ratio (human *i*NKT cells), B (upper and lower pannel) IL4/IFNγ and IFNγ/IL4 ratio (mice *i*NKT cells); two mice per group were injected with 1 μg of lipid ip and sera was assayed at 2 h (IL4) or 24 h (IFNγ) by ELISA.

To evaluate the biological activity of compounds OCH9 (**2**), OCH12 (**3**) and OCH15 (**4**), human B cells expressing CD1d (C1R CD1d) were pulsed with different concentrations of lipids and incubated with a human *i*NKT cell line. *i*NKT cell activation (as determined by both IL-4 and IFN-γ secretion in the culture supernatant) elicited by OCH15 (**4**) was comparable to that obtained with α-GalCer **1** (Fig. 2). On the other hand, compounds OCH9 (**2**) and OCH12 (**3**) were found to be weaker stimulants of *i*NKT cells as less IL-4 and IFN-γ were observed. These data clearly indicate that the *i*NKT cells’ activation by the GSLs OCH9 (**2**), OCH12 (**3**) and OCH15 (**4**) correlate with the length of their sphingosine chains, with the longest chain inducing the greatest cytokine response. However, unlike what was previously observed with mouse *i*NKT cells,^27^ the cytokine profile of human *i*NKT cells stimulated by OCH9 (**2**) and OCH12 (**3**) was not as strongly biased towards a Th2 response (Fig. 2A).^14^ Increasing amounts of IFN-γ were observed with increasing concentrations of OCH9 (**2**) and OCH12 (**3**), as opposed to what was observed with mouse *i*NKT cells (Fig. 3).^27^

We previously performed kinetic and affinity experiments to investigate the effect of the phytosphingosine chain length on the stability of the bound glycolipid/CD1d complex and on TCR binding affinity.^14^ We reported that shortening the phytosphingosine chain increased the rate of dissociation of the GSLs from hCD1d molecules and led to a decrease of the binding affinity of the *i*NKT TCR for hCD1d–GSL complexes.^14^ The increase in the *K*_d_ value as the phytosphingosine chain got shorter was attributed to both a decrease in *k*_on_ and an increase in *k*_off_. Furthermore, our results indicated an important role of the phytosphingosine chain in controlling the formation of the immunological synapse and *i*NKT cell activation.^14^

**Figure 4:**
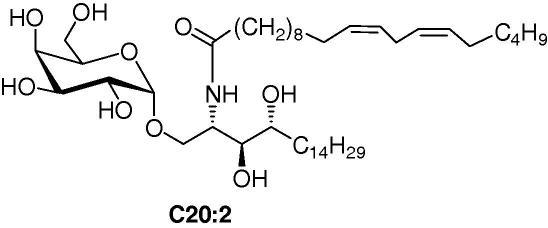
Analogue of α-GalCer inducing a Th2 skewing.

As a control, α-GalCer, OCH9, OCH12 and OCH15 were compared with C20:2 (Fig. 4), a compound known to induce a Th2 skewing in mice.^9^, ^10^, While no apparent change in IL4/IFNγ ratio was observed between α-GalCer and the various GSLs with human *i*NKT cells (Fig. 3A, upper panel), a net increase was observed with mice *i*NKT cells (Fig. 3B, upper pannel). In mice C20:2 and the truncated GSLs OCH9, OCH12 and OCH15 favour the release of the Th2-cytokine IL4. Similarly, the IFNγ/IL4 ratio observed with α-GalCer with mice *i*NKT cells (Fig. 3B, lower pannel) demonstrates that the latter favours the release of the Th1 cytokine IFNγ, confirming that in mice, compounds C20:2, OCH9, OCH12 and OCH15 induce varying extents of Th2 skewing.

## 3 Conclusions

In conclusion we have developed a synthetic strategy for the successful syntheses of three truncated analogues of α-GalCer, adapted from examples present in the literature. ELISA assays of these compounds with hCD1d and human *i*NKT cells showed different results than those observed with mice *i*NKT cells. The truncated analogues OCH9 (**2**), OCH12 (**3**) and OCH15 (**4**) were found to be generally less potent than α-GalCer (**1**). Also there was no Th2 skewing observed in this present study with the shorter phytosphingosine chain lengths. Furthermore, control comparison experiments showed that GSLs OCH9 (**2**), OCH12 (**3**), OCH15 (**4**) and C20:2 cause a Th2 skewing in mice *i*NKT cells, thereby confirming the viability of our assays.

## 4 Experimental

^1^H NMR spectra were recorded at 400 MHz or 300 MHz, using Bruker AMX 400, Bruker AV 400, Bruker AV 300 and Bruker AC 300 spectrometers. ^13^C NMR spectra were recorded at 100 MHz or 75 MHz, respectively, using Bruker AMX 400, Bruker AV 400, Bruker AV 300 and Bruker AC 300 spectrometers. Chemical shifts are reported as *δ* values (ppm) referenced to the following solvent signals: CHCl_3_, *δ*_H_ 7.26; CDCl_3_, *δ*_C_ 77.0; CH_3_OH, *δ*_H_ 3.34; CD_3_OD, *δ*_C_ 49.9. Quaternary carbons were reported only when observed. Optical rotations were measured at 23 °C and reported in degdm^−1^ g^−1^ cm^3^. HRMS were recorded on a Micromass LCT spectrometer using a lock mass incorporated into the mobile phase. All reagents were obtained from commercial sources, and were used without further purification unless stated otherwise. Anhydrous solvents were purchased from Sigma–Aldrich, UK, stored over 4 Å molecular sieves and under an Ar atmosphere. Analytical thin layer chromatography (TLC) was performed on aluminium plates pre-coated with Merck silica gel 60A F-254 as adsorbent. The developed plates were air dried, visualised by UV detection (at 254 nm) and/or stained with 5% phosphomolybdic acid in EtOH (MPA spray). Compounds were purified by flash column chromatography on Merck silica gel (particle size 40–63 lm mesh) or Fluka 60 (40–60 lm mesh) silica gel.

### 4.1 General procedure for the synthesis of phosphonium salts (a)

A mixture of triphenyl phosphine (3 equiv) and the alkyl halide, 1-iodobutane, 1-bromoheptane and 1-bromodecane, respectively, (1 equiv) in toluene was refluxed overnight. The mixture was then allowed to cool and then filtered. The precipitate was washed with cold toluene and dried under vacuo to give the phosphonium salts which were used in the next step without further purification.

### 4.2 General procedure for the synthesis of Wittig salts (ylids) and subsequent olefination (b)

The phosphonium salt was resuspended in THF and cooled to −10 °C. *n*BuLi (2.5 M solution in hexanes, 2.8 equiv) was added dropwise and the reaction mixture was stirred for 30 min, before a solution of 6 (1 equiv) in dry THF was added. The resulting solution was stirred overnight at room temperature. Upon completion of the reaction, the reaction was quenched with MeOH, followed by 80% MeOH in H_2_O, and the mixture was extracted with heptane (4 × 20 mL). The combined organic layers were then washed with brine (30 mL), dried over anhydrous Na_2_SO_4_ and concentrated in vacuo. The residue was purified by purified by flash chromatography using hexanes/EtOAc (10:1) to give the desired products.

### 4.3 (2*R*,3*S*,4*R*,5*E*/*Z*)-3,4-*O*-Isopropylidene-1-*O*-trityloxy-non-5-en-2-ol **7**

Prepared following general procedures (a) and (b) using triphenylphosphine (18.36 g, 70.0 mmol) and iodobutane (4.29 g, 23.3 mmol), *n*-BuLi (26.8 mL, 65.2 mmol) and **6** (10.10 g, 23.3 mmol) to afford compound **7** as a white solid in 82% yield as a mixture of *cis* and *trans* isomer in a 1:2.3 ratio (9.03 g, 19.1 mmol). ^1^H NMR (CDCl_3_): *δ* 7.19–7.49 (15H, m, Ar–H), 5.53–5.57 (2H, m, H-5, H-6), 4.90–4.94 (0.7 H, m, H-4^trans^), 4.45 (0.3H, dd, *J*_4,5_ = *J*_3,4_ = 7.4 Hz, H-4^cis^), 4.28–4.30 (0.3H, m, H-3^cis^), 4.22–4.25 (0.7H, m, H-3^trans^), 3.77–3.81 (0.7H, m, H-2^trans^), 3.69–3.73 (0.3H, m, H-2^cis^), 3.25 (0.3H, dd, *J*_1a,2_ = 5.1, *J*_1a,1b_ = 9.5 Hz, H-1a^cis^), 3.18–3.20 (0.7H, m, H-1a^trans^), 3.07–3.15 (1H, m, H-1b^trans^, H-1b^cis^), 1.75–2.06 (2H, m, H-7a^cis^, H7b^cis^, H-7a^trans^, H-7b^trans^), 1.58, 1.52, 1.39, 1.37 (6H, 4s, 4 × C(CH_3_)_2_), 1.22–1.32 (2H, m, CH_2_), 0.87 (3H, t, *J* = 7.3 Hz, CH_3_); ^13^C NMR (75 MHz, CDCl_3_): *δ* 144.1 (O_2_C(CH3)_2_), 135.8 (C-5), 127.3, 128.0 (C_Ar_), 125.5 (C-6), 79.5 (C-2), 73.3 (C-3), 69.4 (C-4), 65.2 (C-1), 30.6, 30.1 (C-7, C-8), 28.5, 25.7 (2 × C(CH_3_)_3_), 14.9 (C-9); HRMS calcd for C_31_H_36_O_4_ [M+Na]^+^: 495.2511, found 495.2511.

### 4.4 (2*R*,3*S*,4*R*,5*E*/*Z*)-3,4-*O*-Isopropylidene-1-*O*-trityloxy-dodec-5-en-2-ol **8**

Prepared following general procedures (a) and (b) using triphenylphosphine (18.00 g, 68.7 mmol) and 1-bromoheptane (12.24 g, 22.9 mmol), *n*-BuLi (26.4 mL, 64.2 mmol) and **6** (9.90 g, 22.9 mmol) to afford compound **8** as a colourless syrup in 63% yield as a mixture of *cis* and *trans* isomer in a 1:2.3 ratio (5.88 g, 13.68 mmol). ^1^H NMR (CDCl_3_): *δ* 7.19–7.48 (15H, m, Ar–H), 5.47–5.58 (2H, m, H-5, H-6), 4.91–4.93 (0.7 H, m, H-4^trans^), 4.43–4.46 (0.3H, m, H-4^cis^), 4.25 (0.3H, dd, *J*_2,3_ = *J*_3,4_ = 4.6 Hz, H-3^cis^), 4.21 (0.7H, dd, *J*_2,3_ = *J*_3,4_ = 4.4 Hz, H-3^trans^), 3.68–3.81 (0.7H, m, H-2^trans^), 3.51–3.56 (0.3H, m, H-2^cis^), 3.22 (0.3H, dd, *J*_1a,2_ = 5.1, *J*_1a,1b_ = 5.3 Hz, H-1a^cis^), 3.18 (0.7H, dd, *J*_1a,2_ = 9.5, *J*_1a,1b_ = 5.3 Hz, H-1a^trans^), 3.07–3.17 (1H, m, H-1b^trans^, H-1b^cis^), 1.91–2.09 (2H, m, H-7a^cis^, H7b^cis^, H-7a^trans^, H-7b^trans^), 1.54, 1.49, 1.39, 1.38 (6H, 4s, 4 × C(CH_3_)_2_), 1.19–1.39 (8H, m, CH_2_), 0.88 (3H, t, *J* = 5.7 Hz, CH_3_); ^13^C NMR (75 MHz, CDCl_3_): *δ* 145.9, (O_2_C(CH3)_2_), 137.3 (C-5), 129.1, 129.9, 130.8 (C_Ar_), 127.1 (C-6), 79.5 (C-2), 75.1 (C-3), 71.3 (C-4), 67.1 (C-1), 34.2, 31.3, 31.1, 29.7, 25.0 (C-7–C-12), 29.8, 27.1 (2 × C(CH_3_)_3_), 17.1 (C-12); HRMS calcd for C_34_H_42_O_4_ [M+Na]^+^: 537.2980, found 537.2982.

### 4.5 (2*R*,3*S*,4*R*,5*E*/*Z*)-3,4-*O*-Isopropylidene-1-*O*-trityloxy-pentadec-5-en-2-ol **9**

Prepared following general procedures (a) and (b) using triphenylphosphine (54. 6 g, 0.20 mol, 3 equiv) and 1-bromodecane (15.4 g, 69.5 mmol), *n*-BuLi (74.0 mL, 0.18 mol) and **6** (10.00 g, 23.2 mmol) to afford compound **9** as a colourless syrup in 55% yield as a mixture of *cis* and *trans* isomer in a 1:2.3 ratio (7.12 g, 12.8 mmol). ^1^H NMR (CDCl_3_): *δ* 7.19–7.48 (15H, m, Ar–H), 5.47–5.58 (2H, m, H-5, H-6), 4.91–4.93 (0.7 H, m, H-4^trans^), 4.40–4.46 (0.3H, m, H-4^cis^), 4.25 (0.3H, dd, *J*_2,3_ = *J*_3,4_ = 4.6 Hz, H-3^cis^), 4.21 (0.7H, dd, *J*_2,3_ = *J*_3,4_ = 4.4 Hz, H-3^trans^), 3.72–3.79 (0.7H, m, H-2^trans^), 3.68–3.70 (0.3H, m, H-2^cis^), 3.22 (0.3H, dd, *J*_1a,2_ = 5.1, *J*_1a,1b_ = 5.3 Hz, H-1a^cis^), 3.15 (0.7H, dd, *J*_1a,2_ = 9.5, *J*_1a,1b_ = 5.3 Hz, H-1a^trans^), 3.08–3.13 (1H, m, H-1b^trans^, H-1b^cis^), 1.71–2.03 (2H, m, H-7a^cis^, H7b^cis^, H-7a^trans^, H-7b^trans^), 1.56, 1.47, 1.39, 1.38 (6H, 4s, 4 × C(CH_3_)_2_), 1.11–1.35 (14H, m, CH_2_), 0.87 (3H, t, *J* = 6.7 Hz, CH_3_); ^13^C NMR (75 MHz, CDCl_3_): *δ* 145.7, (O_2_C(CH3)_2_), 137.2 (C-5), 130.6, 129.7, 128.9 (C_Ar_), 126.8 (C-6), 79.5 (C-2), 74.9 (C-3), 70.8 (C-4), 66.5 (C-1), 34.0, 31.3, 31.2, 30.0 (C-7–C-14), 29.9, 27.0 (2 × C(CH_3_)_3_), 16.0 (C-15); HRMS calcd for C_37_H_48_O_4_ [M+Na]^+^: 579.3450, found 579.3454.

### 4.6 General procedure for mesylation reaction (c)

Compounds **7**, **8** and **9** (1 equiv) were, respectively, dissolved in a mixture of anhydrous CH_2_Cl_2_ and dry pyridine (3:1) and cooled to 0 °C. Methanesulfonylchloride (1.6 equiv) was then added dropwise and the reaction mixture stirred for 4 h at room temperature. Upon completion of the reaction, the reaction was quenched with sodium bicarbonate solution, diluted with CH_2_Cl_2_ (20 mL) and washed successively with water (20 mL) and brine (20 mL). The organic layer was dried over anhydrous Na_2_SO_4_ and evaporated under vacuo to give the mesylated compounds **10**, **11** and **12** as thick syrups in quantitative yields.

### 4.7 (2*R*,3*S*,*R*,5*E*/*Z*)-3,4-*O*-Isopropylidene-2-methanesulfonyloxy-1-*O*-trityloxy-non-5-enol **10**

Prepared following general procedure (c) using **7** (6.46 g, 13.7 mmol) and methanesulfonyl chloride (1.8 mL, 22.7 mmol) in a mixture of CH_2_Cl_2_ (45 mL) and pyridine (20 mL) to afford compound **10** (6.13 g, 11.2 mmol) as an off-white solid in 82% yield as a mixture of *cis* and *trans* isomer in a 1:2.3 ratio. ^1^H NMR (CDCl_3_): *δ* 7.21–7.49 (15H, m, Ar–H), 5.89–5.48 (2H, m, H-5, H-6), 4.78–5.07 (1H, m, H-2), 4.34–4.50 (2H, m, H-3, H-4), 3.92–4.26 (2H, m, H-1a, H-1b), 3.14, 3.12 (3H, 2s, OSO_2_CH_3_), 1.85–2.12 (2H, m, H-7a^cis^, H7b^cis^, H-7a^trans^, H-7b^trans^), 1.58, 1.52, 1.39, 1.37 (6H, 4s, 4 × C(CH_3_)_2_), 1.22–1.31 (2H, m, CH_2_), 0.85 (3H, t, *J* = 7.2 Hz, CH_3_); ^13^C NMR (75 MHz, CDCl_3_): *δ* 141.4 (O_2_C(CH3)_2_), 135.3 (C-5), 126.9, 126.8, 126.1 (C_Ar_), 121.9 (C-6), 79.2 (C-2), 74.3 (C-3), 70.2 (C-4), 61.3 (C-1), 37.2 (COSO_2_CH_3_), 34.6, 28.7 (C-7, C-8), 28.5, 25.7 (2 × C(CH_3_)_3_), 14.9 (C-9); HRMS calcd for C_32_H_38_SO_6_ [M+Na]^+^: 573.2287, found 573.2290.

### 4.8 (2*R*,3*S*,4*R*,5*E*/*Z*)-3,4-*O*-Isopropylidene-2-methanesulfonyloxy-1-*O*-trityloxy-dodec-5-enol **11**

Prepared following general procedure (c) using **8** (2.59 g, 5.0 mmol) and methanesulfonyl chloride (0.62 mL, 8.06 mmol) in a mixture of CH_2_Cl_2_ (25 mL) and pyridine (8 mL) to afford compound **11** (2.80 g, 4.7 mmol) as a colourless oil in 94% yield as a mixture of *cis* and *trans* isomer in a 1:2.3 ratio. ^1^H NMR (CDCl_3_): *δ* 7.17–7.52 (15H, m, Ar–H), 5.03–5.52 (2H, m, H-5, H-6), 4.79–4.82 (1H, m, H-2), 4.58–4.75 (2H, m, H-3, H-4), 4.48 (0.7H, dd, *J*_1a,2_ = 5.9, *J*_1a,1b_ = 8.4 Hz, H-1a^trans^), 4.24 (0.3H, dd, *J*_1a,2_ = 6.9, *J*_1a,1b_ = 14.0 Hz, H-1a^cis^), 3.54–3.57 (0.3H, m, H-1b^trans^), 3.45 (0.7H, dd, H-1b^cis^), 2.99, 3.18 (3H, 2s, OSO_2_CH_3_), 1.57–1.82 (2H, m, H-7a^cis^, H7b^cis^, H-7a^trans^, H-7b^trans^), 1.48, 1.41, 1.39, 1.38 (6H, 4s, 4 × C(CH_3_)_2_), 1.10–1.33 (8H, m, CH_2_), 0.85 (3H, t, *J* = 6.7 Hz, CH_3_); ^13^C NMR (75 MHz, CDCl_3_): *δ* 144.8 (O_2_C(CH3)_2_), 137.8 (C-5), 130.2, 129.4, 128.7 (C_Ar_), 125.1 (C-6), 82.5 (C-2), 77.6 (C-3), 73.7 (C-4), 64.6 (C-1), 39.9 (COSO_2_CH_3_), 35.1, 30.9, 30.2 (C-7, C-8, C-9), 29.5, 28.1 (2 × C(CH_3_)_3_), 29.0 (C-10), 24.5 (C-11), 16.8 (C-9); HRMS calcd for C_35_H_44_SO_6_ [M+Na]^+^: 615.2757, found 615.2560.

### 4.9 (2*R*,3*S*,4*R*,5*E*/*Z*)-3,4-*O*-Isopropylidene-2-methanesulfonyloxy-1-*O*-trityloxy-pentadec-5-enol **12**

Prepared following general procedure (c) using **9** (5.39 g, 9.67 mmol) and methanesulfonyl chloride (1.3 mL, 16.1 mmol) in a mixture of CH_2_Cl_2_ (45 mL) and pyridine (15 mL) to afford compound **12** (5.52 g, 8.70 mmol) as an opaque oil in 90% yield as a mixture of *cis* and *trans* isomer in a 1:2.3 ratio. ^1^H NMR (CDCl_3_): *δ* 7.19–7.48 (15H, m, Ar–H), 5.40–5.48 (0.7H, m, H-6^trans^), 5.28–5.37 (0.7H, m, H-5^trans^), 5.20–5.25 (0.3H, m, H-6^cis^), 4.97–5.06 (0.3H, M, H-5^cis^), 4.79–4.85 (0.7H, m, H-2^trans^), 4.72–4.75 (0.7H, m, H-4^trans^), 4.62–4.66 (0.3H, m, H-2^cis^), 4.57–4.61(0.3H, m, H-4^cis^), 4.49 (0.7H, dd, *J*_3,4_ = 8.7, *J*_2,3_ = 5.6 Hz, H-3^trans^), 4.20 (0.3H, dd, *J*_3,4_ = 9.4, *J*_3,2_ = 4.2 Hz, H-3^cis^), 3.45–3.56 (2H, m, H-1a, H-1b), 3.04, 3.10 (3H, 2s, OSO_2_CH_3_), 1.85–2.12 (2H, m, H-7a^cis^, H7b^cis^, H-7a^trans^, H-7b^trans^), 1.59–1.82 (2H, m, H-7), 1.48, 1.47, 1.38, 1.36 (6H, 4s, 4 × C(CH_3_)_2_), 1.08–1.32 (14H, m, CH_2_), 0.88 (3H, t, *J* = 6.5 Hz, CH_3_); ^13^C NMR (75 MHz, CDCl_3_): *δ* 144.3 (O_2_C(CH3)_2_), 137.3 (C-5), 129.7, 128.9, 128.2 (C_Ar_), 124.6 (C-6), 82.0 (C-2), 77.1 (C-3), 73.2 (C-4), 64.1 (C-1), 40.1 (COSO_2_CH_3_), 33.1, 32.4, 30.5, 30.3, 30.2 (C-7, C-14), 26.8, 26.6 (2 × C(CH_3_)_3_), 16.9 (C-15); HRMS calcd for C_38_H_50_SO_6_ [M+Na]^+^: 657.3226, found 657.3223.

### 4.10 General procedure for deprotection and reduction of double bond (d)

The mesylated compounds **10**, **11** and **12** were, respectively, dissolved in a mixture of dry CH_2_Cl_2_ and MeOH (2:1) (20 mL) and concentrated hydrochloric acid (3 mL) was added dropwise and the mixture stirred at room temperature for 2 h, after which time TLC analysis indicated that the reaction was complete. Solid sodium bicarbonate was then added to quench the reaction until the solution was neutral. The mixture was then filtered and the filtrate concentrated. The residue was dissolved again in EtOAc and the organic solution washed consecutively with water (2 × 20 mL), brine (20 mL), dried over anhydrous Na_2_SO_4_ and concentrated in vacuo. The residues were purified by flash chromatography (gradient from to hexanes/EtOAc (4:1) to neat EtOAc) and dissolved in THF (10 mL). Five percentage of Pd–Ba(SO_4_)_2_ (0.1 equiv) was added to the solution and the mixture was stirred under H_2_ overnight, after which time it was filtered through a pad of Celite, which was subsequently washed with CHCl_3_/MeOH (1:1). The combined filtrates were concentrated to yield compounds **13**, **14** and **15**.

### 4.11 (2*R*,3*S*,4*R*)-2-Methanesulfonyloxy-nonane-1,3,4-triol **16**

Prepared following general procedure (d) using **10** (5.06 g, 9.18 mmol) to give **16** as an off-white wax (1.91 g, 7.07 mmol) in 77% yield. ^23^[α]_D_ = −80.0 (*c* 1.00, CH_3_OH). ^1^H NMR (CD_3_OD): *δ* 4.84–4.88 (1H, m, H-2), 3.78–3.88 (2H, m, H1a, H-1b), 3.49–3.55 (1H, m, H-4), 3.43–3.47 (1H, dd, *J*_2,3_ = 8.3, *J*_3,4_ = 2.2 Hz, H-3), 3.15 (3H, s, OSO_2_CH_3_), 2.76 (3H, br s, OH), 1.67–1.75 (1H, m, H-5a), 1.46–1.53 (1H, m, H-7a), 1.19–1.38 (6H, m, H-5b, H-6a, H-6b, H-8a, H-8b), 0.88 (3H, t, *J* = 6.8 Hz, CH_3_); ^13^C NMR (75 MHz, CD_3_OD): *δ* 82.9 (C-2), 73.6 (C-3), 70.5 (C-4), 62.3 (C-1), 38.4 (COSO_2_CH_3_), 33.1, 32.0, 25.2, 22.8 (C-5–C-8), 14.0 (C-9); HRMS calcd for C_10_H_22_SO_6_ [M+Na]^+^: 293.1035, found 293.1026.

### 4.12 (2*R*,3*S*,4*R*)-2-Methanesulfonyloxy-dodecane-1,3,4-triol **17**

Prepared following general procedure (d) using **11** (2.46 g, 4.15 mmol) to give **17** as a colourless syrup (0.946 g, 3.03 mmol) in 73% yield. ^23^[α]_D_ = −32.2 (*c* 1.00, CH_3_OH). ^1^H NMR (CD_3_OD): *δ* 4.74–4.78 (1H, m, H-2), 3.62–3.78 (2H, m, H1a, H-1b), 3.31–3.47 (1H, m, H-4), 3.18–3.21 (1H, m, H-3), 3.06 (3H, s, OSO_2_CH_3_), 1.48–1.56 (2H, m, H-5a, H-7a), 1.22–1.43 (12H, m, CH_2_), 0.73 (3H, t, *J* = 6.5 Hz, CH_3_); ^13^C NMR (75 MHz, CD_3_OD): *δ* 83.9 (C-2), 74.7 (C-3), 71.5 (C-4), 63.3 (C-1), 40.1 (COSO_2_CH_3_), 34.2, 33.1, 30.9, 30.8 30.5, 26.5, 23.8 (C-5–C-11), 15.1 (C-12); HRMS calcd for C_13_H_28_SO_6_ [M+Na]^+^: 335.1504, found 335.1511.

### 4.13 (2*R*,3*S*,4*R*)-2-Methanesulfonyloxy-pentadecane-1,3,4-triol **18**

Prepared following general procedure (d) using **12** (6.93 g, 10.9 mmol) to give **18** as a white solid (2.79 g, 7.87 mmol) in 72% yield. ^23^[α]_D_ = −180.0 (*c* 1.00, CH_3_OH). ^1^H NMR (CD_3_OD): *δ* 4.99–5.05 (1H, m, H-2), 3.98–4.04 (2H, m, H1a, H-1b), 3.55–3.62 (1H, m, H-4), 3.45–3.49 (1H, m, H-3), 3.15 (3H, s, OSO_2_CH_3_), 1.48–1.86 (2H, m, H-5a, H-7a), 1.22–1.43 (18H, m, CH_2_), 0.89 (3H, t, *J* = 6.8 Hz, CH_3_); ^13^C NMR (75 MHz, CD_3_OD): *δ* 85.4 (C-2), 75.6 (C-3), 72.8 (C-4), 64.1 (C-1), 40.1 (COSO_2_CH_3_), 34.3, 33.1, 32.0, 30.8, 30.4, 29.2, 28.7, 26.2, 25.2, 22.8 (C-5–C-14), 16.2 (C-15); HRMS calcd for C_16_H_34_SO_6_ [M+Na]^+^: 377.1974, found 377.1980.

### 4.14 General procedure for displacement of mesylate group with sodium azide (e)

The mesylated compounds were dissolved in DMF (10 mL) and sodium azide (2 equiv) was added. The reaction was stirred overnight at 60 °C, after which, it was taken up in water (50 mL) and extracted with EtOAc (3 × 15 mL). The combined organic layers were then washed with brine (30 mL), dried over anhydrous Na_2_SO_4_ and evaporated. The residue was then purified by flash chromatography using hexanes/EtOAc (4:1).

### 4.15 (2*S*,3*S*,4*R*)-2-Azido-nonane-1,3,4-triol **19**

Prepared following general procedure (e) using **16** (0.74 g, 2.75 mmol), sodium azide (0.36 g, 5.50 mmol) to afford a white solid (0.38 g, 1.73 mmol, 63%). ^23^[α]_D_ = +144.0 (*c* 1.00, CH_3_OH). ^1^H NMR (CD_3_OD): *δ* 3.82–3.86 (1H, dd, *J*_1a,1b_ = 11.5, *J*_1a,2_ = 4.9 Hz, H-1a), 3.71–3.77 (1H, m, H-1b), 3.58–3.64 (2H, m, H-3, H-4), 3.44–3.47 (1H, ddd, *J*_1b,2_ = 9.9, *J*_2,3_ = 4.5 Hz), 1.45–1.56 (2H, m, H-5a, H-7a), 1.38 (1H, m, H-5b), 1.27–1.14 (5H, m, CH_2_), 0.79 (3H, t, *J* = 6.1 Hz, CH_3_); ^13^C NMR (75 MHz, CD_3_OD): *δ* 74.0 (C-3), 71.9 (C-4), 62.7 (C-2), 61.0 (C-1), 31.5 (C-5), 31.3 (C-6), 24.9 (C-7), 22.1 (C-8), 13.5 (C-9); HRMS calcd for C_9_H_19_N_3_O_3_[M+Na]^+^: 240.1324, found 240.1315.

### 4.16 (2*S*,3*S*,4*R*)-2-Azido-dodecane-1,3,4-triol **20**

Prepared following general procedure (e) using **17** (0.95 g, 3.03 mmol), sodium azide (0.39 g, 6.06 mmol) to afford a colourless syrup (0.69 g, 2.64 mmol, 87%). ^23^[α]_D_ = +45.0 (*c* 1.00, CH_3_OH). ^1^H NMR (CD_3_OD): *δ* 3.88–3.95 (1H, dd, *J*_1a,1b_ = 11.1, *J*_1a,2_ = 3.3 Hz, H-1a), 3.71–3.80 (1H, m, H-1b), 3.54–3.63 (1H, m, H-4), 3.48–3.53 (1H, m, H-3), 3.28–3.32 (1H, m, H-2), 1.20–1.45 (11H, m, CH_2_), 0.89 (3H, t, *J* = 6.2 Hz, CH_3_); ^13^C NMR (75 MHz, CD_3_OD): *δ* 75.9 (C-3), 72.8 (C-4), 66.2 (C-2), 62.4 (C-1), 33.8, 32.9, 30.7, 30.3, 26.6, 23.6 (C-5–C-11), 13.8 (C-12); HRMS calcd for C_12_H_25_N_3_O_3_[M+Na]^+^: 282.1794, found 282.1794.

### 4.17 (2*S*,3*S*,4*R*)-2-Azido-pentadecane-1,3,4-triol **21**

Prepared following general procedure (e) using **18** (2.64 g, 7.45 mmol), sodium azide (0.97 g, 14.91 mmol) to afford a colourless oil (1.61 g, 5.36 mmol, 72%).^23^[α]_D_ = +31.4 (*c* 1.00, CH_3_OH). ^1^H NMR (CD_3_OD): *δ* 3.92–3.98 (1H, dd, *J*_1a,1b_ = 11.4, *J*_1a,2_ = 3.3 Hz, H-1a), 3.74–3.88 (1H, m, H-1b), 3.58–3.66 (1H, m, H-4), 3.41–3.58 (1H, m, H-3), 3.32–3.36 (1H, m, H-2), 1.51–1.76(3H, m, CH_2_), 1.18–1.47 (17H, m, CH_2_), 0.91 (3H, t, *J* = 6.6 Hz, CH_3_); ^13^C NMR (75 MHz, CD_3_OD): *δ* 73.9 (C-3), 70.8 (C-4), 64.6 (C-2), 60.4 (C-1), 31.8, 31.0, 28.7, 28.4, 24.7, 21.7 (C-5–C-14), 13.6 (C-15); HRMS calcd for C_12_H_25_N_3_O_3_[M+Na]^+^: 324.2263, found 324.2275.

### 4.18 Preparation of glycosyl acceptors **22**, **23** and **24**

Compounds **19**, **20** and **21** were subjected to the same standard reaction conditions described by Akimoto et al.^21^ and depicted in Scheme 2 for the preparation of (2*R*, 3*S*, 4*R*)-2-azido-3, 4-di-*O*-(benzoyloxy)-octadecan-1-ol.

### 4.19 (2*S*,3*S*,4*R*)-2-Azido-3,4-di-*O*-(benzoyloxy)-nonan-1-ol **22**

^23^[α]_D_ = +81.4 (*c* 1.00, CHCl_3_). ^1^H NMR (CDCl_3_): *δ* 7.48–8.07 (10H, m, Ar–H), 5.49–5.46 (2H, m, H-3, H-4), 3.98–4.01 (1H, m, H-1a), 3.75–3.82 (2H, m, H-1b, H-2), 1.82–1.99 (2H, m, H-5a, H-5b), 1.18–1.51 (6H, m, CH_2_), 0.89 (3H, t, *J* = 6.8 Hz, CH_3_); ^13^C NMR (75 MHz, CDCl_3_): *δ* 166.1, 165.7 (C=O), 133.7–129.7 (C_Ar_), 129.3, 129.1, 128.6, 128.4 (C_Ar_), 73.4 (C-3), 72.9 (C-4), 63.1 (C-2), 62.1 (C-1), 31.5 (C-7), 29.7 (C-5), 25.2 (C-6), 22.4 (C-8), 13.9 (C-9); HRMS calcd for C_23_H_27_N_3_O_5_[M+Na]^+^: 448.1848, found 448.1829.

### 4.20 (2*S*,3*S*,4*R*)-2-Azido-3,4-di-*O*-(benzoyloxy)-dodecan-1-ol **23**

^23^[α]_D_ = +8.8 (*c* 1.00, CHCl_3_). ^1^H NMR (CDCl_3_): *δ* 7.26–7.89 (10H, m, Ar–H), 5.32–5.39 (2H, m, H-3, H-4), 3.65–3.82 (1H, m, H-1a), 3.54–3.60 (2H, m, H-1b, H-2), 1.62–1.82 (2H, m, H-5a, H-5b), 1.06–1.30 (12H, m, CH_2_), 0.68 (3H, t, *J* = 6.7 Hz, CH_3_); ^13^C NMR (75 MHz, CDCl_3_): *δ* 136.4.7–131.1 (C_Ar_), 76.1 (C-3), 75.4 (C-4), 66.2 (C-2), 64.8 (C-1), 34.5, 33.0, 30.7, 30.3, 26.6, 23.6 25.3 (C-5–C11), 16.7 (C-12); HRMS calcd for C_26_H_33_N_3_O_5_[M+Na]^+^: 490.2318, found 490.2316.

### 4.21 (2*S*,3*S*,4*R*)-2-Azido-3,4-di-*O*-(benzoyloxy)-pentadecan-1-ol **24**

^23^[α]_D_ = +157.2 (*c* 1.00, CHCl_3_). ^1^H NMR (CDCl_3_): *δ* 7.25–8.11 (10H, m, Ar–H), 5.48–5.5.4 (2H, m, H-3, H-4), 4.82 (1H, dd, *J*_1a,2_ = 7.3, *J*_1a,1b_ = 14.6 Hz, H-1a), 3.67–4.03 (2H, m, H-1b, H-2), 177–1.97 (2H, m, H-5a, H-5b), 1.09–1.46 (18H, m, CH_2_), 0.85 (3H, t, *J* = 6.6 Hz, CH_3_); ^13^C NMR (75 MHz, CDCl_3_): *δ* 162.5 (C=O), 135.8–130.6 (C_Ar_), 75.4 (C-3), 75.1 (C-4), 65.3 (C-2), 64.3 (C-1), 34.0, 32.6, 32.4, 31.5, 29.7, 25.2, 23.8, 23.2, 22.8 22.4 (C-5–C14), 16.2 (C-15); HRMS calcd for C_29_H_39_N_3_O_5_[M+Na]^+^: 532.6370, found 532.6373.

### 4.22 General procedure for glycosylation reaction (f)

To a solution of the persilylated galactose (3 equiv) in anhydrous CH_2_Cl_2_ (20 mL), iodotrimethylsilane (3 equiv) was added and the reaction mixture stirred at room temperature under argon for 30 min. The mixture was then concentrated and azeotroped twice with dry benzene (5 mL). The yellow residue was dissolved in dry benzene and kept under argon. Meanwhile, activated 4 Å molecular sieves, tetrabutyl ammonium iodide (6 equiv), respective glycosyl acceptors **22**, **23** and **24** (1 equiv) and diisopropylethylamine (4.5 equiv) were added to a two-necked flask fitted with a condenser. Benzene (10 mL) was added and the solution stirred at 70 °C for 20 min. The solution of the glycosyl iodide **25** in benzene was then cannulated into the two-necked flask and stirred at room temperature for two days. Upon completion of the reaction, the mixture was filtered through Celite and the Celite pad was washed with CH_2_Cl_2_ (10 mL). The filtrate was washed with saturated sodium thiosulfate (10 mL), brine (10 mL), dried over anhydrous Na_2_SO_4_ and concentrated in vacuo. The residue was dissolved in MeOH and Dowex 50WX8-200 ion exchange resin was added and the mixture stirred at room temperature overnight. The latter was then filtered and the filtrate concentrated to give a residue which was purified by flash chromatography using EtOAc/hexanes (7:1) to give the glycosylated product.

### 4.23 (2*S*,3*S*,4*R*)-2-Azido-3,4-di-*O*-(benzoyloxy)-1-(α-d-galactopyranosyl)-nonane **26**

Prepared following general procedure (f) using per-*O*-pentamethylsilyl-α-d-galactose (0.27 g, 0.49 mmol), iodotrimethylsilane (0.09 g, 0.07 mL, 0.49 mmol), TBAI (3.65 mg, 0.99 mmol), DIPEA (97.5 mg, 0.13 mL, 0.74 mmol) and **22** (70 mg, 0.16 mmol) to afford **26** as a pale yellow oil (43 mg, 0.07 mmol) in 46% yield. ^23^[α]_D_ = +36.1 (*c* 1.00, CHCl_3_). ^1^H NMR (CDCl_3_): *δ*7.38–7.96 (10H, m, Ar–H), 5.59–5.61 (1H, m, H-3^Cer^), 5.40–5.50 (1H, m, H-4^Cer^), 4.80 (1H, s, H-1), 4.10–4.13 (1H, dd, *J*_6a,__6b_ = 7.9, *J*_5,6a_ = 3.0 Hz, H-6a), 3.91–3.94 (2H, m, H-2^Cer^, H-4), 3.80–3.84 (1H, dd, *J*_2,3_ = *J*_3,4_ = 6.0 Hz, H-3), 3.70–3.75 (4H, m, H-2, H-5, H-1a^Cer^, H-1b^Cer^), 3.62–3.67 (1H, m, H-6b), 1.81–1.91 (2H, m, H-5a^Cer^, H-5b^Cer^), 1.15–1.41 (6H, m, CH_2_), 0.83 (3H, t, *J* = 6.4 Hz, CH_3_); ^13^C NMR (75 MHz, CDCl_3_): *δ* 167.1, 167.0 (C=O), 127.4–133.4 (C_Ar_), 101.4 (C-1), 73.2 (C-4^Cer^), 72.1 (C-3^Cer^), 70.9 (C-3), 70.6 (C-5), 70.2 (C-4), 69.7 (C-2), 68.1 (C-6), 62.4 (C-1^Cer^), 61.0 (C-2^Cer^), 21.9–33.3 (4 × CH_2_^Cer^), 13.9 (CH_3_^Cer^); HRMS calcd for C_29_H_37_N_3_O_10_[M+Na]^+^: 610.6183, found 610.6188.

### 4.24 (2*S*,3*S*,4*R*)-2-Azido-3,4-di-*O*-(benzoyloxy)-1-(α-d-galactopyranosyl)-dodecane **27**

Prepared following general procedure (f) using per-*O*-pentamethylsilyl-α-d-galactose (0.85 g, 1.58 mmol), iodotrimethylsilane (0.32 g, 0.23 mL, 1.58 mmol), TBAI (1.17 g, 3.16 mmol), DIPEA (1.05 g, 1.42 mL, 8.16 mmol) and **23** (0.25 g, 0.53 mmol) to afford **27** as an off-white solid (110 mg, 0.17 mmol) in 33% yield.. ^23^[α]_D_ = +92.4 (*c* 1.00, CHCl_3_). ^1^H NMR (CDCl_3_): *δ*7.38–7.98 (10H, m, Ar–H), 5.61–5.64 (1H, m, H-3^Cer^), 5.48–5.52 (1H, m, H-4^Cer^), 4.79 (1H, s, H-1), 4.11–4.13 (1H, dd, *J*_6a,__6b_ = 8.0, *J*_5.6a_ = 2.9 Hz, H-6a), 3.90–3.93 (2H, m, H-2^Cer^, H-4), 3.82–3.85 (1H, m, H-3), 3.71–3.75 (4H, m, H-2, H-5, H-1a^Cer^, H-1b^Cer^), 3.63–3.67 (1H, dd, *J*_5,6b_ = 6.0 Hz, H-6b), 1.81–1.94 (2H, m, H-5a^Cer^, H-5b^Cer^), 1.18–1.42 (12H, m, CH_2_), 0.79 (3H, t, *J* = 6.7 Hz, CH_3_); ^13^C NMR (75 MHz, CDCl_3_): *δ* 166.8, 166.3 (C=O), 128.7–134.2 (C_Ar_), 100.2 (C-1), 73.7 (C-4^Cer^), 72.6 (C-3^Cer^), 71.4 (C-3), 70.6 (C-5), 70.2 (C-4), 69.5 (C-2), 67.9 (C-6), 62.1 (C-1^Cer^), 61.2 (C-2^Cer^), 22.9–32.1 (6 × CH_2_^Cer^), 14.2 (CH_3_^Cer^); HRMS calcd for C_32_H_43_N_3_O_10_[M+Na]^+^: 652.2846, found 610.2845.

### 4.25 (2*S*,3*S*,4*R*)-2-Azido-3,4-di-*O*-(benzoyloxy)-1-(α-d-galactopyranosyl)-pentadecane **28**

Prepared following general procedure (f) using per-*O*-pentamethylsilyl-α-d-galactose (1.61 g, 2.97 mmol), iodotrimethylsilane (0.59 g, 0.43 mL, 2.97 mmol), TBAI (2.19 g, 5.94 mmol), DIPEA (0.58 g, 0.78 mL, 4.45 mmol) and **24** (0.50 g, 0.99 mmol) to afford **28** as an off-white solid (175 mg, 0.26 mmol) in 26% yield.. ^23^[α]_D_ = +81.4 (*c* 1.00, CHCl_3_). ^1^H NMR (CDCl_3_): *δ*7.37–8.02 (10H, m, Ar–H), 5.62–5.66 (1H, m, H-3^Cer^), 5.48–5.55 (1H, m, H-4^Cer^), 4.80 (1H, d, *J*_1,2_ = 2.4 Hz H-1), 4.12–4.16 (1H, m, H-6a), 3.90–3.95 (2H, m, H-2^Cer^, H-4), 3.83–3.85 (1H, m, H-3), 3.71–3.74 (4H, m, H-2, H-5, H-1a^Cer^, H-1b^Cer^), 3.63–3.69 (1H, m, H-6b), 1.82–1.92 (2H, m, H-5a^Cer^, H-5b^Cer^), 1.15–1.30 (16H, m, CH_2_), 0.82 (3H, t, *J* = 6.9 Hz, CH_3_); ^13^C NMR (75 MHz, CDCl_3_): *δ* 135.1–139.5 (C_Ar_), 106.5 (C-1), 79.5 (C-4^Cer^), 78.1 (C-3^Cer^), 71.4 (C-3), 70.8 (C-5), 70.0 (C-4), 69.5 (C-2), 67.7 (C-6), 63.4 (C-1^Cer^), 62.6 (C-2^Cer^), 22.9–36.8 (11 × CH_2_^Cer^), 19.5 (CH_3_^Cer^); HRMS calcd for C_35_H_49_N_3_O_10_[M+Na]^+^: 694.7686, found 694.7683.

### 4.26 General procedure for the removal of benzoate esters, hydrogenation of the azido group and subsequent *N*-acylation (g)

Compounds **26**, **27** and **28** were, respectively, dissolved in MeOH and a 1 M solution of NaOMe (5 mL) was added. The mixture was stirred at room temperature for 2 h, after which time TLC analysis indicated that the reaction was complete. The reaction was neutralised by the addition of Dowex 50WX8-200 resin. The latter was then filtered and the filtrate concentrated to give a residue which was purified by flash chromatography using EtOAc/MeOH (7:1) to give compounds **29**, **30** and **31** in quantitative yields. Compounds **29**, **30** and **31** were, respectively, dissolved in MeOH (10 mL) and stirred with Pd–C (5 mg) under H_2_ overnight, after which time the mixture was filtered through a pad of Celite, which was subsequently washed with MeOH. The combined filtrates were concentrated to give the respective amines as white solids. The crude amine was then dissolved in a 1:1 mixture of THF/NaOAC (saturated solution) (5 mL) and the freshly prepared acid chloride of hexacosanoic acid was added. The reaction was allowed to stir vigorously overnight at room temperature after which the organic phase was removed. The aqueous phase was further extracted with THF (2 × 1 mL), and the combined organic phases were concentrated. The residue was finally purified by flash chromatography (gradient from CHCl_3_ to 15% MeOH in CHCl_3_) to give target compounds **2**, **3** and **4**.

### 4.27 (2*S*,3*S*,4*R*)-1-(α-d-Galactopyranosyl)-2-hexacosanoylamino-3,4-nonanediol **2**

Prepared following general procedure (g) using **26** (50 mg, 0.12 mmol) and hexacosanoic acid (71 mg, 0.18 mmol, 1.5 equiv) to give **2** as a white solid (31 mg, 0.04 mmol) in 35% yield.. ^23^[α]_D_ = +12.6 (*c* 1.00, CHCl_3_/CH_3_OH 2:1). ^1^H NMR (CDCl_3_/CD_3_OD 2:1): *δ* 4.85 (1H, d, *J*_1,2_ = 3.4 Hz, H-1), 4.13–4.16 (1H, m, H-2^Cer^), 3.86–3.89 (1H, m, H-3), 3.81–3.86 (1H, dd, *J*_1a,2_ = 4.6, *J*_1a,1b_ = 10.5 Hz, H-1a^Cer^), 3.72–3.78 (2H, m, H-2, H-5), 3.66–3.72 (3H, m, H-4, H-6a, H-6b), 3.60–3.66 (1H, m, H-1b^Cer^), 3.47–3.55 (2H, m, H-3^Cer^, H-4^Cer^), 2.13–2.18 (2H, m, NHCOCH_2_C_24_H_49_), 1.53–1.67 (3H, m, CH_2_), 0.94–1.46 (49H, m, CH_2_), 0.79 (6H, t, *J* = 6.7 Hz, CH_3_); ^13^C NMR (75 MHz, (CDCl_3_/CD_3_OD 2:1)): *δ* 103.6 (C-1), 77.7 (C-3^Cer^), 75.4 (C-4^Cer^), 73.7 (C-5), 73.2 (C-4), 72.6 (C-3), 71.7 (C-2), 70.7 (C-1^Cer^), 65.2 (C-6), 64.7 (C-2^Cer^), 23.8–33.4 (CH_2_), 15.9 (CH_3_, CH_3_^Cer^); HRMS calcd for C_41_H_81_NO_9_ [M+Na]^+^: 754.5809, found 754.5812.

### 4.28 (2*S*,3*S*,4*R*)-1-(α-d-Galactopyranosyl)-2-hexacosanoylamino-3,4-dodecanediol **3**

Prepared following general procedure (g) using **27** (100 mg, 0.16 mmol) and hexacosanoic acid (95 mg, 0.24 mmol, 1.5 equiv) to give **3** as a white solid (45 mg, 0.06 mmol) in 37% yield. ^23^[α]_D_ = +15.6 (*c* 1.00, CHCl_3_/CH_3_OH 2:1). ^1^H NMR (CDCl_3_/CD_3_OD 2:1): *δ* 4.85 (1H, d, *J*_1,2_ = 3.8 Hz, H-1), 4.12–4.16 (1H, m, H-2^Cer^), 3.87–3.89 (1H, m, H-3), 3.81–3.85 (1H, dd, *J*_1a,2_ = 4.8, *J*_1a,1b_ = 10.5 Hz, H-1a^Cer^), 3.72–3.77 (2H, m, H-2, H-5), 3.66–3.72 (3H, m, H-4, H-6a, H-6b), 3.61–3.66 (1H, m, H-1b^Cer^), 3.47–3.51 (2H, m, H-3^Cer^, H-4^Cer^), 2.14–2.18 (2H, m, NHCOCH_2_C_24_H_49_), 1.46–1.65 (3H, m, CH_2_), 1.16–1.34 (57H, m, CH_2_), 0.82 (6H, t, *J* = 6.6 Hz, CH_3_); ^13^C NMR (75 MHz, (CDCl_3_/CD_3_OD 2:1)): *δ* 101.8 (C-1), 76.9 (C-3^Cer^), 74.2 (C-4^Cer^), 73.2 (C-5), 73.0 (C-4), 72.6 (C-3), 71.8 (C-2), 70.1 (C-1^Cer^), 65.5 (C-6), 64.4 (C-2^Cer^), 24.8–36.0 (CH_2_), 17.0 (CH_3_, CH_3_^Cer^); HRMS calcd for C_44_H_87_NO_9_ [M+Na]^+^: 797.6289, found 797.6291.

### 4.29 (2*S*,3*S*,4*R*)-1-(α-d-Galactopyranosyl)-2-hexacosanoylamino-3,4-pentadecanediol **4**

Prepared following general procedure (g) using **28** (100 mg, 0.15 mmol) and hexacosanoic acid (90 mg, 0.22 mmol, 1.5 equiv) to give **4** as a white solid (44 mg, 0.05 mmol) in 36% yield. ^23^[α]_D_ = +26.7 (*c* 1.00, CHCl_3_/CH_3_OH 2:1). ^1^H NMR (CDCl_3_/CD_3_OD 2:1): *δ* 4.85 (1H, d, *J*_1,2_ = 3.7 Hz, H-1), 4.13–4.16 (1H, m, H-2^Cer^), 3.87–3.89 (1H, m, H-3), 3.81–3.86 (1H, dd, *J*_1a,2_ = 4.8, *J*_1a,1b_ = 11.0 Hz, H-1a^Cer^), 3.72–3.77 (2H, m, H-2, H-5), 3.66–3.71 (3H, m, H-4, H-6a, H-6b), 3.61–3.66 (1H, m, H-1b^Cer^), 3.46–3.51 (2H, m, H-3^Cer^, H-4^Cer^), 2.13–2.18 (2H, m, NHCOCH_2_C_24_H_49_), 1.46–1.65 (3H, m, CH_2_), 1.11–1.35 (70H, m, CH_2_), 0.83 (6H, t, *J* = 6.8 Hz, CH_3_); ^13^C NMR (75 MHz, (CDCl_3_/CD_3_OD 2:1)): *δ* 102.5 (C-1), 76.8 (C-3^Cer^), 74.9 (C-4^Cer^), 73.7 (C-5), 73.1 (C-4), 72.9 (C-3), 71.9 (C-2), 70.7 (C-1^Cer^), 65.0 (C-6), 64.5 (C-2^Cer^), 25.6–35.1 (CH_2_), 16.9 (CH_3_, CH_3_^Cer^); HRMS calcd for C_50_H_99_NO_9_ [M+Na]^+^: 838.6760, found 838.6755.

### 4.30 Elisa

C1R-hCD1d cells were pulsed for overnight with GSLs. 5 × 10^4^ cell-pulsed targets were incubated at 37 °C with 2 × 10^4^ *i*NKT cells in a final volume of 200 μL. After 36 h, the supernatants were harvested, and the concentrations of IFN-γ and IL-4 were determined by commercial ELISA (BD Pharmingen).
